# Background EEG Connectivity Captures the Time-Course of Epileptogenesis in a Mouse Model of Epilepsy

**DOI:** 10.1523/ENEURO.0059-19.2019

**Published:** 2019-08-12

**Authors:** Piotr Słowiński, Laurent Sheybani, Christoph M. Michel, Mark P. Richardson, Charles Quairiaux, John R. Terry, Marc Goodfellow

**Affiliations:** 1 College of Engineering, Mathematics and Physical Sciences, University of Exeter, Exeter, EX4 4QF, United Kingdom; 2 Translational Research Exchange @ Exeter (TREE), University of Exeter, Exeter, EX4 4QD, United Kingdom; 3 Functional Brain Mapping Lab, Department of Fundamental Neuroscience, Campus Biotech, University of Geneva, Geneva, 1202, Switzerland; 4 Centre for Biomedical Imaging (CIBM), Lausanne and Geneva, Lausanne, 1015, Switzerland; 5 Institute of Psychiatry, Psychology and Neuroscience, King's College London, London, WC2R 2LS, United Kingdom; 6 Department of Fundamental Neuroscience, Faculty of Medicine, Geneva, 1206, Switzerland; 7 Centre for Biomedical Modelling and Analysis, University of Exeter, Exeter, EX4 4QD, United Kingdom; 8 EPSRC Centre for Predictive Modelling in Healthcare, University of Exeter, Exeter, EX4 4QD, United Kingdom

**Keywords:** epilepsy, background EEG, model, epileptogenesis, functional networks

## Abstract

Large-scale brain networks are increasingly recognized as important for the generation of seizures in epilepsy. However, how a network evolves from a healthy state through the process of epileptogenesis remains unclear. To address this question, here, we study longitudinal epicranial background EEG recordings (30 electrodes, EEG free from epileptiform activity) of a mouse model of mesial temporal lobe epilepsy. We analyze functional connectivity networks and observe that over the time course of epileptogenesis the networks become increasingly asymmetric. Furthermore, computational modelling reveals that a set of nodes, located outside of the region of initial insult, emerges as particularly important for the network dynamics. These findings are consistent with experimental observations, thus demonstrating that ictogenic mechanisms can be revealed on the EEG, that computational models can be used to monitor unfolding epileptogenesis and that both the primary focus and epileptic network play a role in epileptogenesis.

## Visual Abstract

**Figure:**
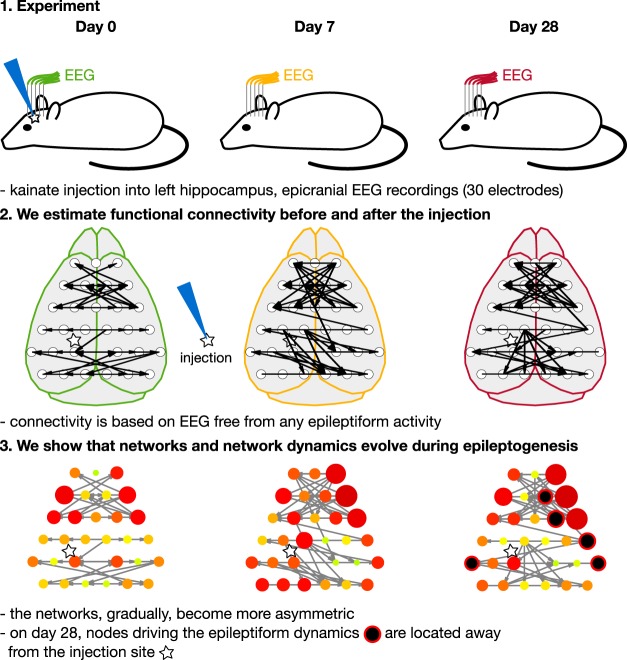

## Significance Statement

We provide the first description of how functional connectivity and network dynamics inferred from background EEG evolve during epileptogenesis. We focus on background EEG because it allows for direct comparison of functional networks before and after experimental intervention. We show that network dynamics inferred by means of computational modeling are different at early and later stages of epileptogenesis. Our findings provide further support for clinical potential of background EEG.

## Introduction

Epilepsy is the most common chronic brain disorder affecting ∼1 in 100 people worldwide and accounting for 0.6% of the global burden of disease (World Health Organization, 2019). Epilepsy is characterized by recurrent seizures. Seizure recurrence is a particularly important feature because up to 10% of people worldwide who do not have epilepsy have a single seizure during their lifetime (World Health Organization, 2019). In other words, although every brain is able to generate seizures, not every brain is prone to generating recurring seizures.

Occurrences of epileptiform activity are irregular and unpredictable, but in contrast background brain activity (i.e., periods of activity that are free from obvious epileptiform abnormalities or discharges) is readily observable. There is therefore a significant research effort focused on exploiting the background activity in research and clinical practice. Recent developments in this area, based on the modern, network perspective of epilepsy, have focused on functional network analyses of background EEG and MEG. These studies have revealed altered networks in the background EEG of people with epilepsy compared with healthy controls (Chowdhury et al., 2014; Schmidt et al., 2014, 2016; Coito et al., 2015; Niso et al., 2015; Woldman and Terry, 2015; Soriano et al., 2017) and have uncovered specific features that can help point to the location of an “epileptogenic zone” within networks (van Dellen et al., 2014; Englot et al., 2015; Nissen et al., 2017). The studies are predominantly concerned with uncovering differences between the EEG of people with epilepsy and healthy controls, and address the question of how ictogenic mechanisms manifest in the EEG. The latter are mechanisms that lead the brain of someone with epilepsy to sporadically transition into seizures from the non-seizure state.

However, a key question in epilepsy research that remains is how does the brain becomes capable of generating recurrent seizures in the first place? This is a question of epileptogenic mechanisms, i.e., what changes does the brain undergo over longer periods of time to become ictogenic (Dichter, 2009; Lopes Da Silva et al., 2012; Goldberg and Coulter, 2013; Löscher et al., 2015). Various animal models can be used to explore these mechanisms. Gill et al. (2017), for example, studied a rat model of intraperitoneally administered kainic acid and catalogued the development of alterations to networks derived from fMRI (Gill et al., 2017). However, our understanding of the ways that large-scale brain dynamics evolve following local insult remains poor.

To address this, we study background functional EEG networks in a well-established mouse model of temporal lobe epilepsy (Bouilleret et al., 1999; Riban et al., 2002; Arabadzisz et al., 2005; Gröticke et al., 2008; Häussler et al., 2012; Lévesque and Avoli, 2013). In this model, unilateral injection of kainic acid in the dorsal hippocampus induces a status epilepticus followed by gradual neurodegeneration at the injected hippocampus (Riban et al., 2002; Arabadzisz et al., 2005). Concomitantly, spontaneous epileptiform events can be measured on the EEG at both hippocampi and after 2–8 weeks, spontaneous and recurrent paroxysmal discharges that are reminiscent of focal and secondarily generalized seizures occur (Riban et al., 2002; Arabadzisz et al., 2005; Chauvière et al., 2012; Huneau et al., 2013; Salami et al., 2014; Sheybani et al., 2018).

In the current study, we characterize functional connectivity networks before and during epileptogenesis by analyzing EEG recorded before kainic acid injection as well as at 7 and 28 days after the injection. Our analysis reveals that the progression of epileptogenesis is reflected in changes to background functional connectivity networks, with the focal injection leading to a disruption of network symmetry. We use a mathematical model to understand how these observed changes affect the ways that different nodes contribute to generation of epileptiform activity. Using only the background activity as input to the model, it reveals that nodes outside of the injected hippocampus become more important throughout epileptogenesis. This is in line with previous experiments that demonstrated the emergence of epileptiform activity self-sustained by brain structures outside of the epileptic focus (the injected hippocampus; Sheybani et al., 2018). These findings present a step toward a network level understanding of epileptogenesis that could be developed to aid diagnosis and treatment of epilepsy.

## Materials and Methods

### Animals and recordings

We used longitudinal recordings from the experiments described by Sheybani et al. (2018). We analyzed longitudinal recordings from 12 animals (adult male C57BL/6J mice, Charles River Laboratories) for which data were recorded before unilateral kainic acid injection into the left hippocampus (Day 0) as well as at 7 and 28 d after injection. Of the 12 longitudinal datasets 1 was excluded from all analysis because of poor quality of the data. Of the 11 remaining datasets 4 were excluded from analysis at Day 7 because of high number of artefacts and noise in the background EEG. Therefore, we used a total of 11 datasets with recordings at Day 0 and Day 28, with 7 of the 11 datasets also including recordings at Day 7. Additionally, we analyzed data recorded from four sham control animals (adult male C57BL/6J mice, Charles River Laboratories) that were unilaterally saline injected into the left hippocampus and had epicranial EEG recorded 28 days after the injection.

The epicranial EEG was recorded at 4 kHz sampling frequency using Digital Lynx SX (NeuraLynx). All recordings were re-referenced to the electrode average. We removed power line interference using a 50 Hz (and 100 and 150 Hz harmonics) notch filter and further bandpass filtered the data between 1 and 150 Hz using a zero-phase forward and reverse Butterworth filter of order 2.

From each EEG recording, which lasted around 30 min, multiple 1 s background data segments were selected from periods without epileptiform activity (median number of segments 55, min 17, max 83); for data collected on Days 7 and 28 the segments were at least 1 s removed from the onset of a generalized spike (GS; inter-ictal epileptic discharges described in Sheybani et al., 2018).

All experiments described by Sheybani et al. (2018) were conducted in accordance with Swiss Laws on animal experimentation.

### Network reconstruction

Following Rummel et al. (2015), Goodfellow et al. (2016), and Schmidt et al. (2016), we treated each EEG channel as recording from a single node of a network. To estimate weights of directed connections between the nodes we combined methods presented by Rummel et al. (2015) and Schmidt et al. (2016). Namely, to measure statistical interdependency between the EEG channels we employed the cross-correlation function:

(1) $$\begin{array}{l} r(x_{i},x_{j})(\tau )=\{\begin{array}{c} \overset{T-\tau }{\underset{t=1}{\sum }}x_{i}(t+\tau )x_{j}(t),\;\tau \geq 0,\\ \overset{T-\left|\tau \right|}{\underset{t=1}{\sum }}x_{i}(t)x_{j}(t+\left|\tau \right|),\;\tau <0, \end{array}\\ r_{coeff}(x_{i},x_{j})(\tau )=\frac{r(x_{i},x_{j})(\tau )}{\sqrt{r(x_{i},x_{i})(0)r(x_{j},x_{j})(0)}}. \end{array}$$

In practice, we used the MATLAB function: xcorr with option coeff, which normalizes the cross-correlation function in such a way that the autocorrelations at 0 lag are equal to 1.

To estimate the strength of the relationship between channels we used three different approaches based on the extremum of the cross-correlogram *r*_coeff_(*x_i_, x_j_*)(*τ*). In the first method, we followed Schmidt et al. (2016), and we used the maximum absolute value of the cross-correlogram, max_τ_ |*r*_coeff_(*x_i_, x_j_*)(*τ*)|. In the second method, we followed suggestion from Sinha et al. (2017) and used only the values of *max*_τ_ |*r*_coeff_(*x_i_, x_j_*)(*τ*)| for which *r*_coeff_(*x_i_, x_j_*)(*τ*)>0. We refer to the matrices derived with these two methods as *C^ABS^* and *C^MAX^*, respectively. Finally, to understand the difference between the *C^ABS^* and *C^MAX^* we also analyzed networks estimated using the values of *max*_τ_ |*r*_coeff_(*x_i_, x_j_*)(*τ*)| where *r*_coeff_(*x_i_, x_j_*)(*τ*)<0. We refer to the matrices from the third method as *C^MIN^*.

The cross-correlogram *r*_coeff_(*x_i_, x_j_*)(*τ*) provides a natural way to infer directionality of the estimated connections. The direction of the connections is given by the sign of the lag between the two channels; with *τ < 0* meaning that a channel *i* is leading (transmitting to) channel *j*. In the paper we adopted notation in which a connection from channel *i* to *j* is noted as element *c_ij_* of the connectivity matrix. In this convention, extrema of the cross-correlation function at *τ < 0* make up the elements of the matrix that are above the diagonal *j > i* and ones at *τ > 0* are below the diagonal *i > j*. The diagonal is equal to 0 (no self-loops).

We disregarded any lags >250 ms (1000 points) and lags <2 ms (8 time samples). We removed the shortest lags to address the problem of volume conduction, i.e. spurious correlations between the time series because of common sources of activity. Such activity is typically detected at very small values of lag between the time series. We chose 8 samples because they correspond to a single sample at sampling frequency 512 Hz, which is a typical sampling frequency used in clinical acquisition of intracranial EEG.

To increase the accuracy of estimation of the connections, we divided each 1 s data segment into 21 windows (500 ms) with a 25 ms overlap, and we computed connectivity matrices for each window.

We further checked that values of the coefficients were not solely because of the presence of dominant intrinsic channel frequencies. For each 1 s data segment we generated 100 sets of univariate iterative amplitude adjusted Fourier transform surrogates (Schreiber and Schmitz, 1996), each containing 30 channels, generated using 10 iterations. A Wilcoxon rank sum test (with Bonferroni correction for 870 comparisons) was used to test, element-wise, whether coefficients in the 21 windows had a different median than the 2100 surrogate windows. For each 1 s data segment the computed values of cross-correlation coefficients were averaged and normalized in the same way as by Rummel et al. (2015):

(2) $$c_{ij}=\frac{〈c_{ij,data}〉-〈c_{ij,surr}〉}{1-〈c_{ij,surr}〉}s_{ij}.$$

Here, ⟨*c_ij,_*_data_⟩ is the median value of the coefficients from the data, ⟨*c_ij,_*_surr_⟩ is the median value of the coefficients from the surrogate data, *s_ij_ =* 1 if the familywise error rate (FWER) < 0.05 and 0 otherwise. Finally, we averaged the network topologies over all data segments in a recording and normalized the coefficients with the sum of all of the elements of the connectivity matrix. By averaging over multiple segments we aimed to estimate functional connectivity that accounts for complex bidirectional interactions between the brain regions generating the recorded activity.

**Figure 1.**
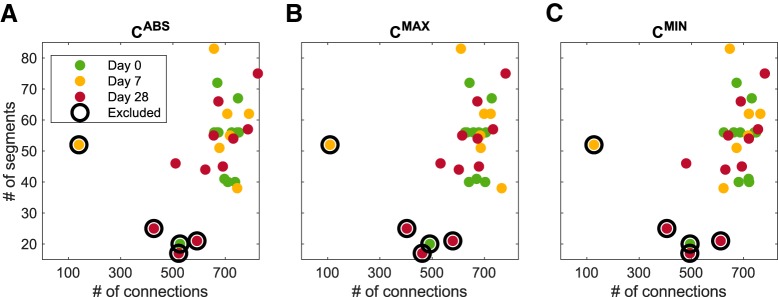
Criteria for excluding recordings from analysis. Number of segments selected in a recording versus number of non-zero elements in the average adjacency matrix estimated from all the segments in the recording. Each dot represents a single recording. Recordings represented by the encircled dots were excluded from the analysis. ***A***, Matrices estimated using *C*^ABS^; ***B***, matrices estimated using *C*^MAX^; ***C***, matrices estimated using *C*^MIN^. Each method produced average adjacency matrices with similar number of non-zero elements.

To ensure that the variability in the number of data segments did not affect the presented results, we excluded from analysis five datasets that either had a very low number of data segments or resulted in a low number of connections (Fig. 1).

### Model

To model the network dynamics we followed the procedure presented by Lopes et al. (2017, 2018), i.e., we analyzed to what extent removal of a single node (virtual resection; Goodfellow et al., 2016; Khambhati et al., 2016) affects activity of the network that on average spends half of the time in the active state. The simulations proceeded as follows:

(1) The dynamics of each node was modelled using the theta neuron model (Ermentrout and Kopell, 1986), which has been shown to well approximate the predictions of neural mass models close to a saddle-node on invariant circle bifurcation (Lopes et al., 2017):

(3) $$\begin{array}{c} \frac{d\theta }{dt}=1-\cos\theta +(1-\cos\theta )I(t),\\ I(t)=I_{0}+I_{n}{}_{oise}N(0,1). \end{array}$$

Here, *I*_0_ is the intrinsic model parameter, *I*_noise_ = 6 is noise intensity and N(0,1) is a random number from a normal distribution with mean 0 and variance 1. We set *I*_0_ *=* −1.2 to ensure that in the absence of noise a stable steady state exists in the system. To couple the nodes, we used the functional connectivity matrix *C*; with elements *c_ij_*. Coupled equations have the following form (Lopes et al., 2017):

(4) $$\begin{array}{l} \frac{d\theta_{i}}{dt}=1-\cos\theta_{i}+(1-\cos\theta_{i})I_{i}(t),\\ I_{i}(t)=I_{0}+I_{n}{}_{oise}N_{i}(0,1)+\omega \sum_{j=1}^{N}c_{ji}[1-\cos(\theta_{j}-\theta_{j}^{*})]/N. \end{array}$$

Here, ω is a global scaling factor of the weights *c_ji_* of the incoming connections of the node *i*; *N* is the total number of nodes in the network. The θ*_j_ is the steady state of node *j*. Parameters *I*_0_ *=* −1.2 and *I*_noise_ = 6 are the same at each node. For each simulation, we used a time step of 0.01, and the duration of the simulation was 4.0e6 time steps. For more details, see Lopes et al. (2017).

(2) We first estimated the value of ω>0 for which on average the whole network spends 50% of the time in the active state. ω_50_ was estimated in separate simulations (averaged over 10 runs with independent noise realizations). We used the same definition of the node’s active state as in (Lopes et al., 2017). To quantify activity of the whole network we use the brain network ictogenicity (BNI), which is the average time each node spends in the active state (Goodfellow et al., 2016):

(5) $$BNI=\frac{1}{N}\sum_{i=1}^{N}\frac{time\;node\;i\;spent\;in\;active\;state}{duration\;of\;simulations}.$$

(3) We then removed a single node and ran simulations with exactly the same parameters; we normalized the sum in Equation 4 with *N* rather than *N−1* to keep the connection weights exactly the same. We measured the change in the network dynamics by comparing the time spent by the network in the active state before and after removing the node. To this end, we used node ictogenicity (NI) defined by Goodfellow et al. (2016):

(6) $$NI_{i}=\frac{0.5-BNI_{i,post}}{0.5},$$

where *BNI_i,_*_post_ is the BNI estimated after removing node *i* from the network. We repeated each simulation 10 times and took the mean value of the NI over the 10 runs with independent noise realizations.

### Statistical methods

We used nonparametric, median based statistical methods (Kruskal–Wallis, Mann–Whitney Wilcoxon or Kolmogorov–Smirnov tests) throughout. To control for multiple comparison during network reconstruction we used the Bonferroni FWER with a significance level of 0.05 (Benjamini and Hochberg, 1995). To control for multiple comparison in the network analysis we used the Benjamini-Hochberg false discovery ratio (FDR; Benjamini and Hochberg, 1995). Due to small sample sizes we used a significance level of 0.1 for the network analysis. We additionally quantified effect sizes using area under the receiver-operating characteristic (AUROC), which is a nonparametric alternative of the common-language effect size (Hentschke and Stüttgen, 2011). We used this method because it has a simple interpretation:

• AUROC = 0.5 means that the scores in the two groups are identical;

• AUROC = 0 means that all scores in the tested group are smaller than the scores of the control group;

• AUROC = 1 means that all scores in the tested group are larger than the scores of the control group.

All presented significant results have AUROC < 0.2 or > 0.8 meaning that the overlap between the scores in the two groups is <20%. In other words, in 80% of the cases a random score from one group exceeds a random score from the other group (Hentschke and Stüttgen, 2011). For the nonparametric one-way ANOVA analysis (Kruskal–Wallis test) we computed *post hoc* AUROC effect sizes of differences between the groups.

To visualize relationships between individual functional connectivity matrices we first quantified pairwise similarity between them by computing the Frobenius distance (Golub and Loan, 1996) for all pairs of matrices:

(7) $${\left\|A-B\right\|}_{F}=\sqrt{\sum_{i=1}^{n}\sum_{j=1}^{m}{\left(a_{ij}-b_{ij}\right)}^{2}},$$

where *a_ij_* and *b_ij_* are the elements of matrices *A* and *B*. Next, we used classical multidimensional scaling (MDS) to visualize relations captured by the similarity matrix (Borg and Groenen, 2005), using MATLAB (Release 2018b, MathWorks) function cmdscale.

### Statistical table

Description of statistical tests and the significance levels for results in Figs 3 and 4 can be found in Table 1. Description of statistical tests and the significance levels for the other results are described in the text.

### Code accessibility

MATLAB scripts for the network analysis are available on request from P.S. The model is subject to copyright owned by the University of Exeter (international patent application WO/2017029505).

## Results

Our goal is to characterize the evolution of large-scale functional brain networks during epileptogenesis. Many measures exist to quantify functional connectivity (Wang et al., 2014), each with different underlying assumptions. We begin with no *a priori* knowledge regarding the way in which the evolving ictogenic mechanisms of the brain may be reflected in functional connectivity. We therefore do not restrict our analysis to a particular frequency band. Considering broadband signals, a natural way to quantify functional connectivity is to study the correlation between signals. To avoid problems associated with volume conduction, we use the cross-correlation function and exclude correlations with maximum at zero lag (Christodoulakis et al., 2015). Focusing on lagged correlations also gives a natural way to build directionality into the networks. Additionally, the resulting correlations can be positive or negative and there are therefore different ways to quantify strength of interactions in the derived functional network. First, one can quantify the strength of the connection using the maximum of the absolute value of the cross-correlogram (Schmidt et al., 2014). We refer to the networks estimated using this method as *C*^ABS^. Second, one can neglect negative values (Sinha et al., 2017) and use only the values of *C*^ABS^ at which the cross-correlogram *>0*. We refer to networks estimated using this method as *C*^MAX^. To analyze the differences between *C*^ABS^ and *C* ^MAX^ one can also examine the networks derived from the values of *C^ABS^* at which the cross-correlogram *<0*. We refer to these networks as *C*^MIN^. In other words, one can decompose the connectivity matrices *C*^ABS^ into component matrices *C*^MAX^ and *C*^MIN^. See Materials and Methods for details of the reconstruction of the connectivity matrices. In the following sections, we examine functional connectivity through epileptogenesis using these three methods.

### Epileptogenesis changes properties of background functional connectivity networks

**Figure 2.**
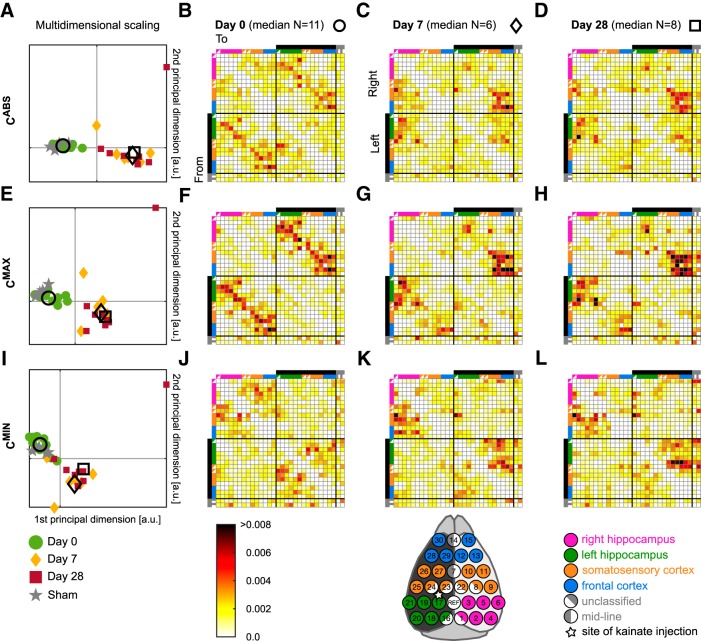
Analysis of background functional connectivity reveals changes over the time course of epileptogenesis. ***A***, ***E***, ***I***, Individual connectivity matrices represented as dots in the first two principal dimensions of the multidimensional scaling of Frobenius distances between the individual connectivity matrices. Each dot represents a single matrix (green, Day 0; yellow, Day 7; red, Day 28; gray, Sham control; empty symbols: circle, diamond, and square represent the median of the connectivity matrices). The first three principal multidimensional scaling dimensions represent ∼70% of the relations encoded in the raw Frobenius distances (*R*^2^_ABS_=0.66, *R*^2^_MAX_=0.72, *R*^2^_MIN_=0.7; *R* is Pearson’s correlation coefficient between the Frobenius distances in the matrix space and the Euclidian distances in the reconstructed space); for clarity only the first two coordinates are plotted. ***B*–*D***, ***F*–*H***, ***J*–*L***, Median functional connectivity matrices (indicated with empty symbols in ***A***, ***E***, ***I***) resulting from the three different measures at different days with color-coded connection weights (Day 0 over 11 matrices, Day 7 over 6 matrices, Day 28 over 8 matrices; different numbers of matrices for individual days because of quality of recordings; see Materials and Methods).

Figure 2 demonstrates the evolution of functional connectivity across the first 4 weeks of epileptogenesis for the three types of networks introduced above. The functional connectivity is described by connectivity matrices: each entry in a connectivity matrix represents a statistical relationship (in this case the extremum of cross-correlogram that occurred for non-zero lag) between EEG signals at two different electrodes. Therefore, the connectivity matrix captures the correlation pattern of a multichannel EEG signal.

We quantified the differences between the connectivity matrices of individual animals across three different time points (Days 0, 7 and 28) by calculating the Frobenius distance between them (see Materials and Methods; Borg and Groenen, 2005). Using these distances to visualize the similarity between the matrices reveals that control (Day 0 and Sham) networks are different to post-injection networks (Days 7 and 28), because they form a distinct cluster compared with matrices derived from recordings at Days 7 and 28 for each of the three measures (Fig. 2*A*,*E*,*I*). The clustering of points corresponding to matrices derived from recordings before and after injection visible in Figure 2*A*, *E*, and *I*, demonstrates that the kainic acid injection has a large and consistent effect on the correlation patterns of the epicranial EEG. The clusters, however, do not inform us about which components of the connectivity matrices have changed.

To study the data on the population level, we compute median correlation matrices for each time point (median over entries *c_ij_* of the connectivity matrices). Figure 2 demonstrates that the median correlation matrices appear to progress from an initially symmetric arrangement at Day 0, to asymmetric networks post-injection (Days 7 and 28). It also shows that the *C*^ABS^ matrices are a composition of the *C*^MAX^ and *C*^MIN^ matrices and that the *C*^MAX^ and *C*^MIN^ matrices differ from each other. A characteristic feature of the *C*^MAX^ networks is that the connections between contralateral regions appear to be among the strongest (Fig. 2*F*–*H*, top right and bottom left quadrants of the connectivity matrices). To quantify the redistribution of connections post-injection, we asked whether connections from each electrode to their contralateral equivalent (dark anti-diagonals of the quadrants) were among the strongest (i.e., in 5% of the strongest connections). For control networks, 38% of contralateral connections were among the strongest, whereas this percentage fell to 22% at Days 7 and 28. This means that post-injection, the EEG between hemispheres becomes less synchronized. We note that this trend was also observed if we considered raw as opposed to normalized connectivity matrices. Such a decrease in synchronization has previously been shown for the hippocampi (Arabadzisz et al., 2005), but not for other brain regions. In contrast, for the *C* ^MIN^ networks the strongest connections are ipsilateral, meaning that they represent connections within a hemisphere (Fig. 2*J*–*L*, top left and bottom right quadrants of the connectivity matrices).

To quantify the breakdown of synchronization, we calculated the degree imbalance (outdegree−indegree) of nodes in the functional connectivity networks of individual animals. Degree imbalance is an aggregated measure that quantifies the strength of connectivity for each node. Statistically, if outdegree_weighted_>indegree_weighted_ the EEG signal recorded on a node temporally leads some of the other nodes and the node can be interpreted as a “source” of activity. If not, the node lags other nodes on average and it can be considered a “sink” (outdegree_weighted_<indegree_weighted_).

**Figure 3.**
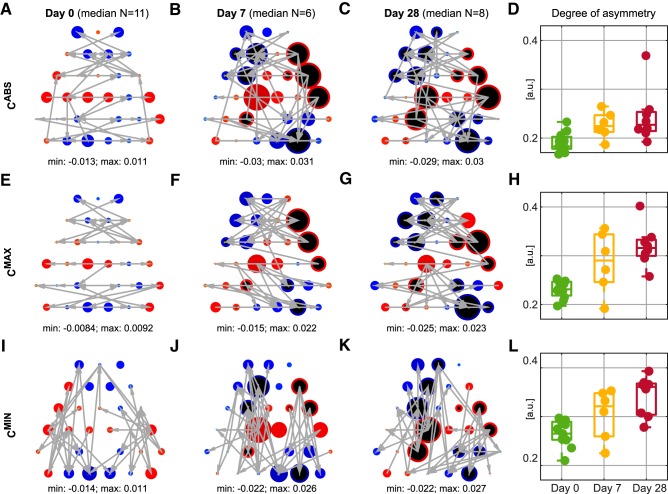
Illustration of changes of network properties over the time course of epileptogenesis. ***A*–*C***, ***E*–*G***, ***I*–*K***, Median degree imbalance at individual nodes; blue indicates indegree>outdegree, red indicates indegree<outdegree. Value of the degree imbalance is color and size coded; larger and darker dots indicate higher degree imbalance. Dots filled in black have a median that is significantly different from the median on Day 0 (FDR < 0.1, two-sided Wilcoxon Mann–Whitney test with Benjamini–Hochberg correction for 30 nodes, effect size AUROC < 0.2 for blue nodes or >0.8 for red nodes; exact *p* values and effect sizes are presented in Extended data Figure 3-1). Gray arrows show topology of functional connectivity networks on different days illustrated using the strongest 5% of connections of the median connectivity matrices shown in Figure 2. ***D***, ***H***, ***L***, Boxplots showing the degree of assymetry of the individual connectivity matrices.

Interestingly, although network topologies are different for each of the three methods considered, the degree imbalance of the *C*^ABS^, *C*^MAX^, and *C*^MIN^ networks are similar. Figure 3*A*–*C*, *E*–*G*, and *I*–*K* shows the distribution of median degree imbalance across nodes. At Day 0, the configuration is symmetric, with sinks (Fig. 3*A*,*E*,*J*, blue nodes) predominantly in anterior and posterior regions. The maximum absolute values of the degree imbalance at Day 0 are approximately two times lower than at Days 7 and 28. At Day 7 the degree imbalance increases, with sources located at the left posterior and the right anterior regions. This pattern persists through to Day 28. Interestingly, many of the nodes that became sources are located above the left hippocampus, i.e., the site of initial intrahippocampal injection (Sheybani et al., 2018).

10.1523/ENEURO.0059-19.2019.f3-1 Figure 3-1 Detailed illustration of changes in spatial distribution of degree imbalance (DI). ***A*–*C***, Boxplots of distributions of DI values on each node on Day 0 (green), Day 7 (yellow) and Day 28 (Red). Shaded yellow bar indicates significant difference between Day 0 and Day 7. Shaded red bar indicates significant difference between Day 0 and Day 28. Text labels are as follows: first row FDR, second row (AUROC) for comparison of Day 0 and Day 28; third row FDR and fourth row (AUROC) for comparison of Day 0 and Day 7. Two-sided Wilcoxon Mann–Whitney test with Benjamini–Hochberg FDR correction for 30 nodes; effect size measured as AUROC. Download Figure 3-1, EPS file.

Figure 3*A*–*C*, *E*–*G*, and *I*–*K* shows the network topology of the top 5% of the strongest connections of the median connectivity matrices presented in Figure 2. These networks corroborate our observations based on the connectivity matrices: *C*^ABS^ matrices are a composition of the *C*^MAX^ and *C*^MIN^ matrices; the strongest connections in the *C*^MAX^ matrices are contralateral and the strongest connections in *C*^MIN^ matrices are ipsilateral. Taken collectively, Figures 2 and 3 describe changes in symmetry of the connectivity matrix and illustrate the large-scale breakdown of synchronization between right and left hemispheres that can be revealed from background EEG through epileptogenesis.

In addition to analyzing the degree imbalance of nodes, we analyzed global properties of the functional connectivity networks (Table 2; Fig. 3*D*,*H*,*L*). For all three types of network the same measures (Spectral norm, Variance of neighbor weighted outdegree and degree of asymmetry) were found to be significantly different on Day 0 and Days 7 and 28 (FDR < 0.05, Kruskal–Wallis test with Benjamini–Hochberg FDR correction for 19 tested network measures; chosen to capture in a nonredundant way the most important topological and spectral properties of the networks; for all analyzed measures, see Table 2). Values of these three measures increase over the time course of epileptogenesis; as an example, Figure 3*D*, *H*, and *L*, illustrate increasing median of the degree of asymmetry (Li and Zhang, 2012). These changes in local and global network properties further indicate that the underlying functional connectivity pattern of background activity becomes progressively more irregular and spatially heterogeneous post injection.

**Table 1.** Statistical table

**Results**	**Data structure**	**Statistical test**	**Power or confidence intervals**
Fig. 3A–*C*, *E*–*G*, *I*–*K*	No assumptions about the distributions of the degree imbalance on each of the 3 days.	Two-sided Wilcoxon Mann–Whitney test with Benjamini–Hochberg multiple comparison/FDR correction for 30 nodes.Separate comparison for: Day 0 vs Day 7 and Day 0 and Day 28.We use two-sided test because we expect to see increase and decrease of degree imbalance.	Panel *B*:FDR ≤ 0.08,AUROC<0.2 or AUROC>0.8;Panel *C*:FDR ≤ 0.08,AUROC<0.2 or AUROC>0.8;Panel *D*:FDR ≤ 0.1,AUROC<0.2 or AUROC>0.8;Panel *E*:FDR ≤ 0.07,AUROC<0.2 or AUROC>0.8;Panel *G*:FDR ≤ 0.07,AUROC<0.2 or AUROC>0.9;Panel *H*:FDR ≤ 0.07,AUROC<0.2. or AUROC>0.9;See Extended data Figure 3-1 for values.
Fig. 3D, *H*, *L*; Table 2	No assumptions about the distributions of the network measures.	The Kruskal–Wallis test (nonparametric ANOVA) with Benjamini–Hochberg multiple comparison/FDR correction for 20 analyzed measures.	FDR and AUROC values are reported in Table 2.
Fig. 4	No assumptions about the distributions of the node ictogenicity on each of the 3 days.	One-sided Wilcoxon Mann–Whitney test with Benjamini–Hochberg multiple comparison/FDR correction for 30 nodes.Separate comparison for: Day 0 vs Day 7 and Day 0 and Day 28.We use one-sided test because only test increase of node icotgenicity.	Panel *B*: FDR ≤ 0.1, AUROC>0.8;Panel *C*: FDR ≤ 0.09, AUROC>0.8;Panel *E*: FDR ≤ 0.08, AUROC>0.8;Panel *G*: FDR ≤ 0.02, AUROC=1;Panel *H*: FDR ≤ 0.01, AUROC<0.9.See Extended data Figure 4-1 for values.

Columns are part of the results section, the structure of the data, statistical test, and description of the significance levels.

**Table 2.** Statistical analysis of network properties for the three kinds of connectivity matrices

**Name of the network property**	***C*^ABS^**	***C*^MAX^**	***C*^MIN^**
Mean weighted outdegree	0.69	0.83	0.5
Variance of weighted outdegree	**0.055** **(**0.23; **0.13)**	**0.035** **(0.17; 0.13)**	0.12
Spectral norm	**0.0099** **(0; 0.091)**	**0.0083** **(0.061; 0.046)**	**0.026** **(0.11; 0.13)**
Frobenius norm	0.42	0.47	0.27
Mean neighbor weighted outdegree	0.21	0.24	0.4
Variance of neighbor weighted outdegree	**0.0099** **(0.03; 0.079)**	**0.0131** **(0.076; 0.1)**	**0.0233** **(**0.71; **0.89)**
Mean betweenness	0.20	0.75	0.66
Variance of betweenness	**0.098** **(0.12;** 0.48**)**	0.44	0.3
Mean page-rank	0.4	0.96	0.58
Variance of page-rank	0.17	0.47	0.27
Mean length of the shortest path between two nodes	0.29	0.47	0.4
Variance of length of the shortest paths	0.17	0.8	0.4
Mean harmonic closeness centrality	0.29	0.47	0.4
Variance of harmonic closeness centrality	**0.068** **(0.91;** 0.62**)**	0.2	0.5
Assortative mixing (Pearson’s total weighted degree correlation)	0.17	0.66	0.058
S-metric, sum of the product of nodal degrees across edges	0.81	0.5	0.78
Degree of asymmetry, largest eigenvalue of the skew-symmetric part of the Laplacian of a directed graph (Li and Zhang, 2012).	**0.026** **(0.14; 0.1)**	**0.011** **(**0.21; **0)**	**0.026** **(**0.26; **0.045)**
Mean spectrum, mean of eigenvalues of symmetric part of the Laplacian matrix of the directed graph (Li and Zhang, 2012).	0.74	0.8	0.14
Variance of spectrum, variance of eigenvalues of symmetric part of the Laplacian matrix of the directed graph (Li and Zhang, 2012).	0.4	0.25	0.88
Maximum of spectrum, largest eigenvalue of symmetric part of the Laplacian matrix of the directed graph (Li and Zhang, 2012).	0.17	0.25	0.76

Table contains values of the Benjamini–Hochberg FDR for the Kruskal–Wallis test for comparison of medians of measures on Days 0, 7, and 28. In brackets *post hoc* effect sizes quantified with AUROC: (Day 0 vs Day 7; Day 0 vs Day 28). Bold values show FDR < 0.1 and AUROC < 0.2 or AUROC > 0.8.

### Epileptogenesis changes network dynamics

**Figure 4.**
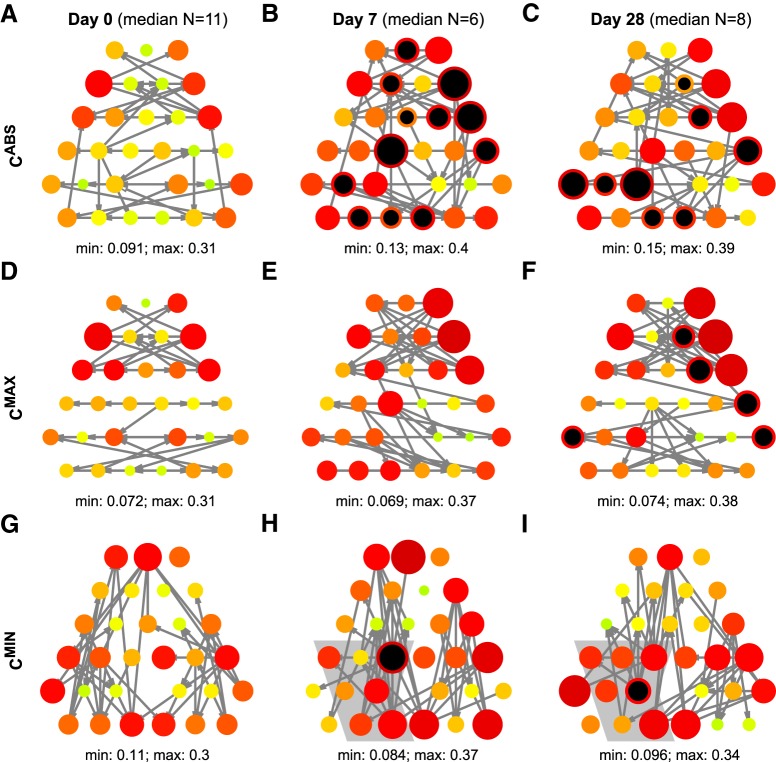
Illustration of changes in spatial distribution of node ictogenicity. ***A*–*I***, Mean values of NI. Gray arrows are the strongest 5% of connections of the median networks. Value of the NI is color and size coded; larger and darker dots indicate higher NI. Dots filled in black have significantly higher median NI than the median on Day 0 (FDR < 0.1 one-sided Wilcoxon Mann–Whitney test with Benjamini–Hochberg FDR correction for 30 nodes, effect size AUROC > 0.8; exact *p* values and effect sizes are presented in Extended data Figure 4-1). Shaded regions in ***H*** and ***I*** show nodes affected by the TTX silencing; identified from Sheybani et al. (2018), their Figure 12*B*.

An important question is how these alterations to the pattern of functional connectivity inferred from background EEG influence the ways that nodes contribute to the generation of epileptiform dynamics. To make this mechanistic link, we studied a mathematical model of spiking dynamics placed on the nodes of networks derived from each animal (see Material and Methods). To measure the contribution that each node in a network has to the generation of epileptiform rhythms we use NI introduced by Goodfellow et al. (2016; see Material and Methods). Figure 4 shows the distribution of NI at Days 0, 7, and 28 for the three types of networks. At Day 0, which we use as a reference point, we see that the NI is distributed symmetrically through the network, but with slightly elevated values in frontal regions. This means that, if the network was ictogenic, nodes in frontal regions would contribute more to the generation of epileptiform dynamics. At Day 7, the *C* ^ABS^ networks, shown in Figure 4*B*, displays significantly higher NI for multiple nodes in the left posterior and right anterior regions. This pattern persists at Day 28 (Fig. 4*C*), though nodes with elevated NI are now constrained to fewer regions. For the *C* ^MAX^ networks, illustrated in Figure 4*E* and *F*, significant increases in NI above baseline only occur at Day 28. Finally, for the *C* ^MIN^ networks, NI increases significantly at a single node, the location of which changes between Days 7 and 28. On both days the node with significantly elevated NI resides within a region that has been shown to be affected by TTX silencing, as identified by Sheybani et al. (2018) (their Figure 12*B*). In the experiments described by Sheybani et al. (2018) the kainate injected hippocampus (left) was silenced using an intrahippocampal TTX injection. After the TTX injection on Day 7, interictal GSs subsided. The same procedure on Day 28 did not affect the frequency of occurrence of GSs.

10.1523/ENEURO.0059-19.2019.f4-1 Figure 4-1 Detailed illustration of changes in spatial distribution of NI. ***A*–*C***, Boxplots of distributions of NI values on each node on Day 0 (green), Day 7 (yellow), and Day 28 (red). Shaded yellow bar indicates significant difference between Day 0 and Day 7. Shaded red bar indicates significant difference between Day 0 and Day 28. Text labels are as follows: first row FDR, second row (AUROC) for comparison of Day 0 and Day 28; third row FDR and fourth row (AUROC) for comparison of Day 0 and Day 7. One-sided Wilcoxon Mann–Whitney test with Benjamini–Hochberg FDR correction for 30 nodes; effect size measured as AUROC. Download Figure 4-1, EPS file.

GSs are interictal epileptic discharges recently reported to be a predominant EEG marker of evolving abnormal dynamics during the latent as well as chronic phase of the disease in the kainic acid model (Sheybani et al., 2018). GSs travel across the whole epileptic network and have also been observed in humans (Aarts et al., 1984; Mohamed et al., 2001; Moseley et al., 2012). Sheybani et al. (2018) showed that the frequency of occurrence of GSs increases during epileptogenesis and that their occurrence is correlated with increased jerky movements. Furthermore, by Day 28 GSs no longer depend on the activity of the injected hippocampus, as captured by the TTX silencing experiment and evolution of the location of their onsets throughout Days 0–28 (Sheybani et al., 2018, their Fig. 6*E*). At Day 7, GSs originate predominantly from the left and right posterior regions, which is reflected in the observed increase in NI in left posterior regions and also node 9 in the *C* ^ABS^ networks. However, changes in NI are also observed in anterior regions in our model results. At Day 28, GSs originate predominantly from the right posterior regions, which is best captured by the evolution of NI in *C* ^MAX^ networks.

## Discussion

Network analyses are increasingly being used to refine diagnosis, prognosis and treatment for epilepsy (Schmidt et al., 2014, 2016; Englot et al., 2015; Niso et al., 2015; Rummel et al., 2015; Tracy and Doucet, 2015; Goodfellow et al., 2016; Smith and Schevon, 2016; Lopes et al., 2017, 2018). In humans, functional connectivity derived from the background EEG are known to be altered in epilepsy. For example, Englot et al. (2015) showed that patients with focal epilepsies (temporal and neocortical) had decreased resting-state functional connectivity in multiple brain regions. In addition, people with idiopathic generalized epilepsies, as well as their first-degree relatives, have been shown to have elevated mean-degree and mean-degree variance of background functional EEG networks (Chowdhury et al., 2014).

Here we have provided the first characterization of how functional connectivity inferred from background EEG evolves during epileptogenesis. Throughout epileptogenesis, functional connectivity networks that are initially regular and symmetric become irregular and asymmetric. This corresponds to a loss of functional connectivity between hemispheres, both in the normalized connectivity presented in Figure 2 and if the raw connectivity is considered. These changes observed using EEG are in line with previous studies of fMRI functional connectivity derived in the tetanus toxin model (Otte et al., 2012), and could be underpinned by changes in white matter tracts (Otte et al., 2012) or changes to dynamics within localized brain regions. However, they differ from the analysis of the fMRI-derived functional connectivity in the systemic kainic acid model of temporal lobe epilepsy, which displayed stronger connections in comparison with control animals (Gill et al., 2017). Potential reasons for these discrepancies include the intraperitoneal administration of kainic acid used by Gill et al. (2017) causing more widespread changes in the brain tissue than intrahippocampal administration. Furthermore, functional networks reported by Gill et al. (2017) were estimated using long duration recordings (tens of minutes vs seconds in our study) from anesthetized animals (awake head-fixed animals in the current study). Additionally, neither of these previous studies addressed the process of epileptogenesis through repeated observations within the same animal.

To relate our findings of altered functional connectivity to the generation of epileptiform activity, we used a mathematical model. The model allowed us to define the relative contribution of nodes to the generation of epileptiform dynamics. Our model showed that the set of nodes that are important for epileptiform dynamics evolves over 4 weeks of epileptogenesis. Two of the three different methods we used to compute functional connectivity network revealed nodes outside of the injected hippocampus that were important contributors to epileptiform dynamics. Specifically, significant changes in the NI distribution of the *C*^MIN^ connectivity networks (at which the cross-correlogram *<0*) capture the increase of NI over the injected hippocampus, which occurs 7 days after the injection and persists through to Day 28. In contrast, the *C*^MAX^ connectivity networks (at which the cross-correlogram *>0*) reveal changes in the distribution of NI only at Day 28, involving multiple nodes that are located outside the injected hippocampus.

We hypothesize that *C*^MIN^ and *C*^MAX^ networks reflect two mechanisms that generate GSs. The first mechanism is local and related to the initial insult (the injected hippocampus), whereas the other mechanism is distributed and is a consequence of network remodeling. Importantly, Figure 4*D*–*F* shows that the second mechanism emerges at a time subsequent to the initial insult. This interpretation is consistent with the results of (Sheybani et al., 2018) in which pharmacological silencing of the injected hippocampus at Day 7 stopped GSs, whereas it had no effect when performed at Day 28. This suggests the evolving importance of a distributed network throughout epileptogenesis. In other words, results of the modeling suggest that the injected hippocampus is driving the epileptiform activity at Day 7, whereas at Day 28 the activity is driven by both the injected hippocampus as well as the wider network.

Additionally, we note that changes in NI across individual nodes are directly interpretable in terms of generation of the GSs and the results of the silencing experiments, whereas typical graph theory measures (e.g., degree imbalance or degree asymmetry) do not allow such direct interpretation. This observation provides further support for the use of mathematical models to uncover regions of the brain that are important for generating abnormal dynamics and to aid the interpretation of experimental and clinical data (Goodfellow et al., 2016; Schmidt et al., 2016; Bartolomei et al., 2017; Hebbink et al., 2017; Lopes et al., 2017, 2018; Melozzi et al., 2017; Proix et al., 2017). A natural next step would be to model the process of epileptogenesis itself to better understand why these changes occur, and why they occur in specific brain regions. Insights into spatial and temporal evolution of epileptogenesis could help to develop new treatments (Dichter, 2009; Lowenstein, 2009; Löscher and Brandt, 2010; Lopes Da Silva et al., 2012; Goldberg and Coulter, 2013; Löscher et al., 2015) and uncover reasons for seizure recurrence after epilepsy surgery (Mathon et al., 2017).

We express caution in relating observations made in this study to human epilepsy, as we expect mouse epicranial EEG contains contributions from brain structures that are subcortical in humans (e.g., hippocampus) and therefore would contribute less to the background human EEG (Gotman, 2008; Lam et al., 2017). The recordings analyzed herein are perhaps more analogous to ECoG or depth electrode recordings in humans. In this scenario, the approach of modeling activity recorded from invasive electrodes has shown promise in predicting the outcome of surgery in people with diverse “focal” epilepsies (Goodfellow et al., 2017; Lopes et al., 2017, 2018; Sinha et al., 2017). Our study advances our understanding of such approaches and demonstrates a framework that allows for their experimental validation.

## Synthesis

Reviewing Editor: Maiken Nedergaard, University of Rochester Medical Center Decisions are customarily a result of the Reviewing Editor and the peer reviewers coming together and discussing their recommendations until a consensus is reached. When revisions are invited, a fact-based synthesis statement explaining their decision and outlining what is needed to prepare a revision will be listed below. The following reviewer(s) agreed to reveal their identity: Piotr Suffczynski, Holger Lerche.

In general, the reviewers appreciated the study and have no major critique. We will, therefore, request that you frevise the manuscript and provide a point-to-point letter that described the requested revisions.

Reviewer #1:

The Ms describes properties of functional networks at different stages of epieleptogenesis in the kainic acid model of temporal lobe epilepsy. Such longitudinal analysis has not been done before.

The Ms concerns connectivity analysis in the kainic acid model of temporal lobe epilepsy. The authors investigate time evolution of functional networks based on resting state EEG kainate-treated mice. They convincingly show progressive changes in pattern of connectivity during epileptogenesis. The study is well written and the results are novel. I suggest acceptance after minor revision.

Main point

The authors observe increased hemispheric asymmetry of the functional networks during epileptogenesis and interpret it as a loss of functional connectivity between hemispheres (pg. 9, ln. 434). The cross-correlation coefficients were normalized with the sum of all elements of the connectivity matrix (pg. 4). Hence, relative decrease of inter-hemispheric coupling may be indeed due to loss of functional connectivity between hemispheres or due to increase of functional connectivity between other areas, e.g., intra-hemispheric, with functional inter-hemispheric coupling preserved. The Authors should discuss this point, especially that some other studies, which used BOLD signals correlations and non-normalized cross-correlation matrices showed increase of both inter- and intra-hemispheric connectivity in kainate-treated rats (Gill et al., 2017).

Minor points:

- Reference at pg 11, ln 525 is: Da Silva FHL, Gorter JA, Wadman WJ (2012) ...., while it should be Lopes da Silva FH, Gorter JA, Wadman WJ (2012) ...... (the surname is Lopes da Silva, the names are Fernando, Henrique).

- pg. 4, abbreviations FWER, IAAFT are used without explanation. They might be not obvious for all readers. Please explain.

- pg. 6, ln247-250:

„AUROC=0 means that all scores in the tested group are smaller than the scores of the control group;

AUROC=1 means that all scores in the tested group are smaller than the scores of the control group.”

The statements are identical. Is it correct?

- Section on pg. 6, ln 286-288 is redundant with that on pg. 3, ln. 135-138.

- TTX silencing is mentioned on pg. 9, ln 406. Please provide short explanation, such that one doesn't need to read the original publication (Sheybani et al., 2018).

References:

Ravnoor Singh Gill, Seyed M. Mirsattari and L. Stan Leung. Resting state functional network disruptions in a kainic acid model of temporal lobe epilepsy. Neuroimage Clin. 2017; 13: 70-81.

Sheybani L, Birot G, Contestabile A, Seeck M, Zoltan Kiss J, Schaller K, Michel CM, Quairiaux C (2018) Electrophysiological evidence for the development of a self-sustained large- scale epileptic network in the kainate mouse-model of temporal lobe epilepsy. Journal of Neuroscience

The reviewer does not believe that the supplementary material is justified

Reviewer #2:

The authors report about the evolution of network dynamics measured with 30 EEG electrodes distributed over the whole brain in a classical mouse model for temporal lobe epilepsy (intrahippocampal kainate injection). The find changes in networks which parallel the development of epileptogenesis. The work is original, since such longitudinal studies have not been performed so far, neither in animal models, nor in humans.

Comments

The authors do not report about which of the two hippocampi was injected and how the evolution matches the development of epileptogensis on the injected side vs. the non-injected side. This is particularly important, since the epileptogenic process is different on both sides and the network changes are changing side with time. It is also not clear, if only the injected hippocampus was silenced at different time points (day 7 or day 28) or also the other one in the preceeding paper from the authors took the data for their analysis. The authors should clearly report this and explicitely correlate the unilateral and asymmetric network changes to the occurrence of spikes on the injected and uninjected side in the respective animals. They so far write only about ‘generalized spikes’ (which in fact represent a fast bilateral synchrony derived from focal spikes), which would be unusual for a unilateral injection process.

The manuscript is generally well written but there are some minor language inaccuracies, which should be solved, best by a native English speaker, some of which are given below:

Line 13/14: should read ‘...emerges as particularly important for the network dynamics.’ (?)

Line 356: should read ‘...located above the left hippocampus...’

Line 370: ‘...networks...’
